# Publisher Correction: Drosophilids with darker cuticle have higher body temperature under light

**DOI:** 10.1038/s41598-023-31605-9

**Published:** 2023-03-21

**Authors:** Laurent Freoa, Luis-Miguel Chevin, Philippe Christol, Sylvie Méléard, Michael Rera, Amandine Véber, Jean-Michel Gibert

**Affiliations:** 1 Laboratoire de Biologie du Développement, UMR 7622, CNRS, Institut de Biologie Paris-Seine (IBPS), Sorbonne Université, 9 Quai St-Bernard, 75005 Paris, France; 2 CNRS, MAP5, Université Paris Cité, 45 Rue des Saints-Pères, 75006 Paris, France; 3 CEFE, CNRS, EPHE, IRD, Univ Montpellier, Univ Paul Valéry Montpellier 3, 34000 Montpellier, France; 4 UMR5214, CNRS, Institut d’électronique et des systèmes, Université de Montpellier, 34000 Montpellier, France; 5 CMAP, CNRS, Ecole Polytechnique, France et Institut Universitaire de France, Institut Polytechnique de Paris, 91120 Palaiseau, France; 6 Inserm UMR U1284, Centre de Recherche Interdisciplinaire (CRI Paris), 8 bis Rue Charles V, 75004 Paris, France

Correction to : *Scientific Reports* 10.1038/s41598-023-30652-6, published online 02 March 2023

The original version of the Article contained an error in the Author Information section, where the authors Laurent Freoa and Luis‑Miguel Chevin were incorrectly tagged as authors who jointly supervised the work.

The original Article has been corrected.

