# Supplementary material for: Drosophilids with darker cuticle have higher body temperature under light
Source: Sci Rep. 2023 Mar 2;13:3513. doi: 10.1038/s41598-023-30652-6 (PMC9981618; doi:10.1038/s41598-023-30652-6)

Drosophilids with darker cuticle have higher body temperature under light

Laurent Freoa. Luis-Miguel Chevin. Philippe Christol. Sylvie Méléard. Michael Rera. Amandine Véber+ and Jean-Michel Gibert+*

+ co-last authors.

* corresponding author: jean-michel.gibert@sorbonne-universite.fr

Supplementary tables and figures:

Table S1: Data set for D. melanogaster ebony mutant females. The unit of measure is in Celsius and rounded down to nearest 0.01.

	**Before inversion**			**After inversion**			**Mean between two inversions**		
In °	Fly	Ellipse	Fly - Ellipse	Fly	Ellipse	Fly - Ellipse	Fly	Ellipse	Fly - Ellipse
**1**	22.30	20.13	2.17	21.92	19.75	2.17	22.11	19.94	2.17
**2**	22.31	20.11	2.20	22.40	20.18	2.22	22.35	20.14	2.21
**3**	22.49	20.48	2.01	22.28	20.24	2.04	22.38	20.36	2.02
**4**	22.57	20.18	2.39	22.75	20.19	2.56	22.66	20.18	2.48
**5**	22.12	20.06	2.06	22.29	20.23	2.06	22.20	20.14	2.06
**6**	22.18	20.06	2.12	22.55	20.08	2.47	22.36	20.07	2.29
**7**	22.15	20.01	2.14	22.22	20.21	2.01	22.18	20.11	2.07
**8**	22.62	20.33	2.29	22.62	20.25	2.37	22.62	20.29	2.33
**9**	21.84	19.69	2.15	21.98	19.84	2.14	21.91	19.76	2.15
**10**	22.38	20.23	2.15	21.68	19.82	1.86	22.03	20.02	2.01
**11**	22.84	20.44	2.40	22.20	20.13	2.07	22.52	20.28	2.24
**12**	23.03	20.43	2.60	22.63	20.16	2.47	22.83	20.29	2.54
**13**	22.54	19.89	2.65	22.69	20.00	2.69	22.61	19.94	2.67
**14**	20.65	18.86	1.79	21.67	19.47	2.20	21.16	19.16	2.00
**15**	23.41	20.40	2.99	22.99	19.80	3.19	23.20	20.10	3.10

Table S2: Data set for *D. melanogaster yellow* mutant females. The unit of measure is in Celsius and rounded down to nearest 0.01.

	**Before inversion**			**After inversion**			**Mean between two inversions**		
In °	Fly	Ellipse	Fly - Ellipse	Fly	Ellipse	Fly - Ellipse	Fly	Ellipse	Fly - Ellipse
**1**	21.42	19.87	1.55	21.23	19.87	1.36	21.32	19.87	1.45
**2**	21.50	19.93	1.57	21.85	20.17	1.68	21.67	20.05	1.62
**3**	21.89	20.26	1.63	22.10	20.25	1.85	21.99	20.25	1.74
**4**	21.80	20.01	1.79	22.04	20.18	1.86	21.92	20.09	1.83
**5**	21.38	19.83	1.55	21.69	20.04	1.65	21.53	19.93	1.60
**6**	21.26	19.94	1.32	21.82	20.20	1.62	21.54	20.07	1.47
**7**	21.68	20.14	1.54	21.63	19.93	1.70	21.65	20.03	1.62
**8**	21.85	20.08	1.77	22.07	20.30	1.77	21.96	20.19	1.77
**9**	21.37	19.73	1.64	21.24	19.71	1.53	21.30	19.72	1.58
**10**	21.79	20.42	1.37	21.32	19.70	1.62	21.55	20.06	1.49
**11**	21.93	20.23	1.70	21.49	20.12	1.37	21.71	20.17	1.54
**12**	22.05	20.28	1.77	22.07	20.06	2.01	22.06	20.17	1.89
**13**	21.37	19.75	1.62	21.60	19.93	1.67	21.48	19.84	1.64
**14**	19.98	18.66	1.32	20.93	19.50	1.43	20.45	19.08	1.37
**15**	22.55	20.21	2.34	21.89	19.88	2.01	22.22	20.04	2.18

Table S3: Data set for *D. americana* females. The unit of measure is in Celsius and rounded down to nearest 0.01.

	**Before inversion**			**After inversion**			**Mean between two inversions**		
In °	Fly	Ellipse	Fly - Ellipse	Fly	Ellipse	Fly - Ellipse	Fly	Ellipse	Fly - Ellipse
**1**	23.71	20.09	3.62	23.83	20.00	3.83	23.77	20.04	3.72
**2**	23.61	20.72	2.89	23.09	20.26	2.83	23.35	20.49	2.86
**3**	24.14	20.39	3.75	23.61	20.25	3.36	23.87	20.32	3.55
**4**	23.97	20.45	3.52	23.19	20.26	2.93	23.58	20.35	3.22
**5**	24.42	20.53	3.89	24.45	20.55	3.90	24.43	20.54	3.89
**6**	24.42	20.88	3.54	24.46	21.06	3.40	24.44	20.97	3.47
**7**	23.57	20.34	3.23	24.54	21.13	3.41	24.05	20.73	3.32
**8**	23.32	19.79	3.53	23.82	20.79	3.03	23.57	20.29	3.28
**9**	23.02	19.96	3.06	22.92	19.97	2.95	22.97	19.96	3.00
**10**	23.45	20.27	3.18	23.28	20.28	3.00	23.36	20.27	3.09
**11**	22.82	20.18	2.64	22.67	19.95	2.72	22.74	20.06	2.68
**12**	23.12	20.12	3.00	22.82	19.93	2.89	22.97	20.02	2.94
**13**	23.30	20.12	3.18	22.90	20.01	2.89	23.10	20.06	3.03

Table S4: Data set for *D. novamexicana* females. The unit of measure is in Celsius and rounded down to nearest 0.01.

	**Before inversion**			**After inversion**			**Mean between two inversions**		
In °	Fly	Ellipse	Fly - Ellipse	Fly	Ellipse	Fly - Ellipse	Fly	Ellipse	Fly - Ellipse
**1**	22.97	20.01	2.96	23.09	20.04	3.05	23.03	20.02	3.00
**2**	23.40	20.93	2.47	22.62	19.86	2.76	23.01	20.39	2.61
**3**	22.95	20.36	2.59	22.55	19.96	2.59	22.75	20.16	2.59
**4**	23.35	20.84	2.51	22.78	19.90	2.88	23.06	20.37	2.69
**5**	23.40	20.63	2.77	23.18	20.15	3.03	23.29	20.39	2.90
**6**	24.39	21.48	2.91	23.73	20.98	2.75	24.06	21.23	2.83
**7**	22.75	20.24	2.51	23.68	20.99	2.69	23.21	20.61	2.60
**8**	22.70	19.92	2.78	23.07	20.35	2.72	22.88	20.13	2.75
**9**	22.62	19.83	2.79	22.24	19.80	2.44	22.43	19.81	2.61
**10**	22.70	20.44	2.26	22.99	20.30	2.69	22.84	20.37	2.47
**11**	22.32	20.23	2.09	22.14	19.87	2.27	22.23	20.05	2.18
**12**	22.43	20.14	2.29	22.21	19.93	2.28	22.32	20.03	2.28
**13**	22.74	20.20	2.54	22.55	19.94	2.61	22.64	20.07	2.57

Table S5: Data set for *D. yakuba* males. The unit of measure is in Celsius and rounded down to nearest 0.01.

	**Before inversion**			**After inversion**			**Mean between two inversions**		
In °	Fly	Ellipse	Fly - Ellipse	Fly	Ellipse	Fly - Ellipse	Fly	Ellipse	Fly - Ellipse
**1**	20.76	19.27	1.49	20.75	19.08	1.67	20.75	19.17	1.58
**2**	20.61	19.08	1.53	20.86	19.14	1.72	20.73	19.11	1.62
**3**	20.72	19.25	1.47	20.80	19.22	1.58	20.76	19.23	1.52
**4**	20.87	19.03	1.84	20.86	19.05	1.81	20.86	19.04	1.82
**5**	20.83	19.30	1.53	20.43	19.02	1.41	20.63	19.16	1.47
**6**	20.61	19.19	1.42	20.75	18.97	1.78	20.68	19.08	1.60
**7**	20.70	19.39	1.31	20.85	19.40	1.45	20.77	19.39	1.38
**8**	20.55	19.37	1.18	20.49	19.26	1.23	20.52	19.31	1.20
**9**	20.73	19.36	1.37	21.03	19.40	1.63	20.88	19.38	1.50
**10**	21.89	19.78	2.11	21.38	19.72	1.66	21.63	19.75	1.88
**11**	21.69	19.93	1.76	21.74	19.63	2.11	21.71	19.78	1.93
**12**	21.92	20.12	1.80	21.80	20.09	1.71	21.86	20.10	1.75
**13**	21.74	20.11	1.63	21.79	19.93	1.86	21.76	20.02	1.74

Table S6: Data set for *D. santomea* males. The unit of measure is in Celsius and rounded down to nearest 0.01.

	**Before inversion**			**After inversion**			**Mean between two inversions**		
In °	Fly	Ellipse	Fly - Ellipse	Fly	Ellipse	Fly - Ellipse	Fly	Ellipse	Fly - Ellipse
**1**	20.64	19.17	1.47	20.57	19.18	1.39	20.60	19.17	1.43
**2**	20.43	19.01	1.42	20.74	19.12	1.62	20.58	19.06	1.52
**3**	20.65	19.21	1.44	20.86	19.26	1.60	20.75	19.23	1.52
**4**	20.73	18.94	1.79	20.78	19.15	1.63	20.75	19.04	1.71
**5**	20.81	19.19	1.62	20.20	19.00	1.20	20.50	19.09	1.41
**6**	20.47	19.11	1.36	20.50	19.05	1.45	20.48	19.08	1.40
**7**	20.76	19.23	1.53	20.80	19.45	1.35	20.78	19.34	1.44
**8**	20.33	19.25	1.08	20.13	19.21	0.92	20.23	19.23	1.00
**9**	20.41	19.29	1.12	20.40	19.43	0.97	20.40	19.36	1.04
**10**	21.47	19.63	1.84	21.47	19.84	1.63	21.47	19.73	1.73
**11**	21.31	19.67	1.64	21.53	19.87	1.66	21.42	19.77	1.65
**12**	21.68	19.89	1.79	21.80	20.23	1.57	21.74	20.06	1.68
**13**	21.60	19.88	1.72	21.60	20.13	1.47	21.60	20.00	1.59

Table S7: Data set for *D. melanogaster Dark* females. The unit of measure is in Celsius and rounded down to nearest 0.01.

	**Before inversion**			**After inversion**			**Mean between two inversions**		
In °	Fly	Ellipse	Fly - Ellipse	Fly	Ellipse	Fly - Ellipse	Fly	Ellipse	Fly - Ellipse
**1**	22.37	20.51	1.86	22.11	20.20	1.91	22.24	20.35	1.88
**2**	21.96	20.18	1.78	21.71	19.85	1.86	21.83	20.01	1.82
**3**	22.10	19.95	2.15	22.35	20.52	1.83	22.22	20.23	1.99
**4**	22.10	19.88	2.22	22.16	19.96	2.20	22.13	19.92	2.21
**5**	21.62	20.21	1.41	20.97	19.28	1.69	21.29	19.74	1.55
**6**	21.50	19.89	1.61	21.77	19.91	1.86	21.63	19.90	1.73
**7**	21.14	19.30	1.84	22.14	20.31	1.83	21.64	19.80	1.83
**8**	21.93	19.94	1.99	21.82	20.00	1.82	21.87	19.97	1.90
**9**	22.01	20.25	1.76	21.26	19.37	1.89	21.63	19.81	1.82
**10**	21.65	20.00	1.65	21.41	19.66	1.75	21.53	19.83	1.70
**11**	21.55	19.74	1.81	21.94	20.18	1.76	21.74	19.96	1.78
**12**	22.05	20.15	1.90	21.34	19.45	1.89	21.69	19.80	1.89
**13**	21.84	20.18	1.66	21.62	19.53	2.09	21.73	19.85	1.87

Table S8: Data set for *D. melanogaster Pale* females. The unit of measure is in Celsius and rounded down to nearest 0.01.

	**Before inversion**			**After inversion**			**Mean between two inversions**		
In °	Fly	Ellipse	Fly - Ellipse	Fly	Ellipse	Fly - Ellipse	Fly	Ellipse	Fly - Ellipse
**1**	22.38	20.66	1.72	21.82	20.09	1.73	22.10	20.37	1.72
**2**	22.05	20.33	1.72	21.51	19.74	1.77	21.78	20.03	1.74
**3**	21.77	20.14	1.63	22.02	20.24	1.78	21.89	20.19	1.70
**4**	21.70	19.24	2.46	21.47	20.17	1.30	21.58	19.70	1.88
**5**	21.62	20.13	1.49	21.13	19.41	1.72	21.37	19.77	1.60
**6**	21.36	19.83	1.53	21.82	20.05	1.77	21.59	19.94	1.65
**7**	20.83	19.15	1.68	22.10	20.29	1.81	21.46	19.72	1.74
**8**	21.64	19.83	1.81	21.73	20.14	1.59	21.68	19.98	1.70
**9**	21.79	20.13	1.66	21.28	19.52	1.76	21.53	19.82	1.71
**10**	21.33	19.99	1.34	21.21	19.69	1.52	21.27	19.84	1.43
**11**	21.29	19.59	1.70	21.96	20.26	1.70	21.62	19.92	1.70
**12**	21.73	20.10	1.63	21.54	19.59	1.95	21.63	19.84	1.79
**13**	21.73	20.03	1.70	21.66	19.74	1.92	21.69	19.88	1.81

Table S9: Data set for the differences in pigmentation (hue) between *D. melanogaster ebony* and *yellow* females

Pair	e	y	e-y
1	122.647	23.136	99.511
2	125.997	23.94	102.057
3	126.885	22.841	104.044
4	119.301	23.362	95.939
5	122.901	23.318	99.583
6	122.86	23.137	99.723
7	137.346	23.284	114.062
8	129.611	22.436	107.175
9	124.849	23.908	100.941
10	134.695	23.28	111.415
av			103.445
sdv			5.766710693

Table S10: Data set for the differences in pigmentation (hue) between *D. americana* and *D. novamexicana* females.

Pair	am	nov	am-nov
1	138.209	60.691	77.518
2	141.134	57.092	84.042
3	134.999	51.507	83.492
4	139.709	53.319	86.39
5	138.133	78.61	59.523
6	137.843	65.907	71.936
7	139.118	49.19	89.928
8	138.903	79.833	59.07
9	139.555	49.702	89.853
10	140.085	59.122	80.963
av			78.2715
sdv			11.37551863

Table S11: Data set for differences in pigmentation (hue) between *D. yakuba* and *D. santomea* males.

Pair	yak	san	yak-san
1	99 .064	40.703	58.361
2	91.602	37.892	53.71
3	75.343	32.904	42.439
4	94.659	37.596	57.063
5	80.121	32.927	47.194
6	90.541	51.593	38.948
7	89.812	32.751	57.061
8	97.771	33.126	64.645
9	94.73	32.19	62.54
10	108.87	31.939	76.931
av			55.8892
sdv			11.15936474

Table S12: Data set for differences in pigmentation (hue) between *D. melanogaster Dark* and *Pale* females

Pair	Dark	Pale	Dark-Pale
1	55.001	33.273	21.728
2	61.057	38.35	22.707
3	70.451	39.265	31.186
4	63.642	38.813	24.829
5	73.008	47.719	25.289
6	65.827	53.597	12.23
7	68.294	46.299	21.995
8	67.578	51.269	16.309
9	64.982	43.25	21.732
10	69.728	47.324	22.404
av			22.0409
std			5.075386235

Figure S1:

a: Histogram of the differences between the normalized temperature of the *D. melanogaster ebony* fly and that of the *D. melanogaster yellow* fly. It is set to have 7 bins. The box on the top-right of the picture is the result of a Wilcoxon signed rank exact test of the symmetry of the distribution of the difference between the two coordinates of data_norm with respect to its mean. The value of the test statistic is V = 57 and the associated p-value is 0.8871. Since this p-value is larger than 0.05. we cannot reject the hypothesis that the distribution of the temperature difference may be considered to be symmetric about its mean. The size of the data set is 15.

b: Histogram of the differences between the normalized temperature of *D. americana* and that of *D. novamexicana*. It is set to have 7 bins. The box on the top-right of the picture is the result of a Wilcoxon signed rank exact test to test the symmetry of the distribution of the difference between the two coordinates of data_norm with respect to its mean. The value of the test statistic is V = 43 and the associated p-value is 0.8926. Since the p-value is larger than 0.05. we cannot reject the hypothesis that the distribution of the temperature difference may be considered to be symmetric about its mean. The size of the data set is 13.

c: Histogram of the differences between the normalized temperature of *D. yakuba* and that of *D. santomea*. It is parametrised to have 7 bins. The box on the top-right of the picture is the result of a Wilcoxon signed rank exact test to test the symmetry of the distribution of the difference between the two coordinates of data_norm with respect to its mean. The value of the test statistic is V = 43 and the associated p-value is 0.8926. Since the p-value is larger than 0.05. we cannot reject the hypothesis that the

distribution of the temperature difference may be considered to be symmetric about its mean. The size of the data set is 13.

d: Histogram of the differences between the normalized temperature of *D. melanogaster Dark* line and that of *D. melanogaster Pale* line. It is set to have 7 bins. The box on the top-right of the picture is the result of a Wilcoxon signed rank exact test to test the symmetry of the distribution of the difference between the two coordinates of data_norm with respect to its mean. The test statistic is V = 42 and the associated p-value is 0.8394. Here again. we cannot reject the hypothesis that the distribution of the temperature difference may be considered to be symmetric about its mean. The size of the data set is 13.

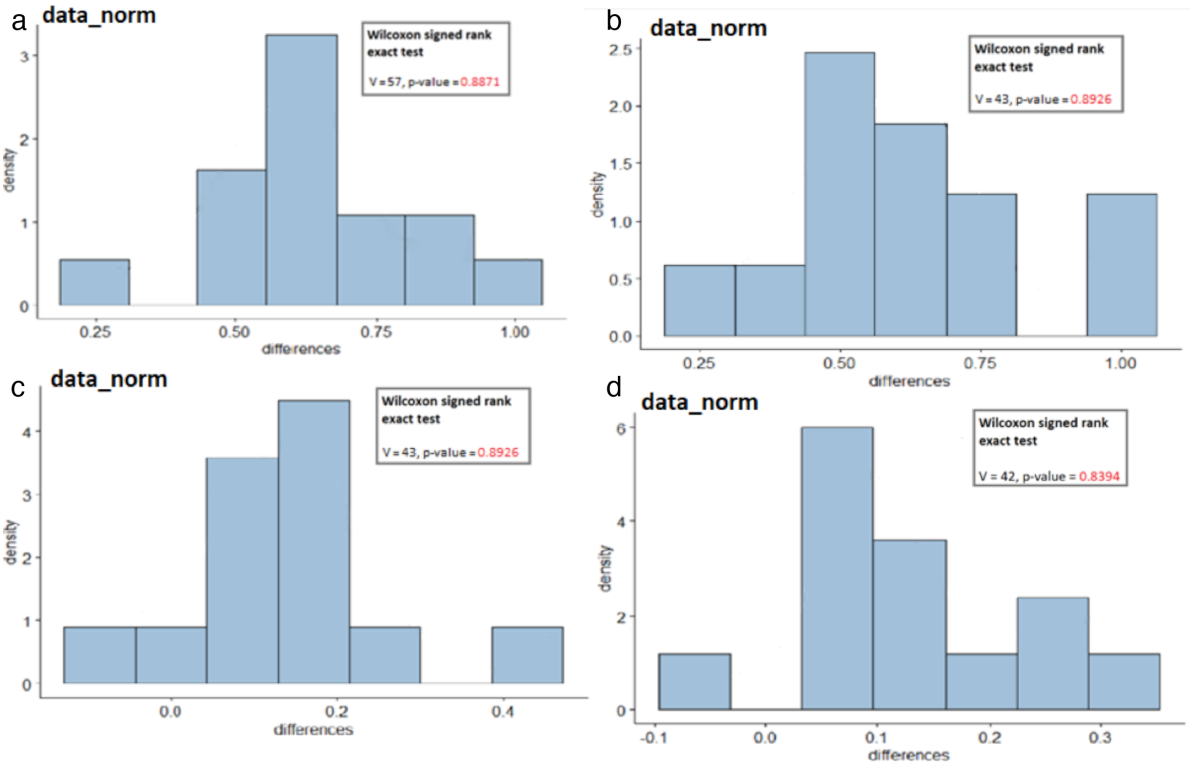

Supplement: Supplementary file 1 — Supplementary Information. [file 41598_2023_30652_MOESM1_ESM.pdf]
